# Simple Effective Ways to Care for Skin Wounds and Incisions

**DOI:** 10.1097/GOX.0000000000002471

**Published:** 2019-10-25

**Authors:** Don Lalonde, Nadim Joukhadar, Jeff Janis

**Affiliations:** From the * Dalhousie University, Saint John, NB, Canada; † Dalhousie University, Halifax, NS, Canada; ‡ Ohio State University Wexner Medical Center, Columbus, Ohio.

## Abstract

Plastic surgeons are medicine’s wound experts. Many of the world’s poor cannot afford expensive wound management programs. All humans suffer open and closed wounds at some point in their life and must look after them. The purpose of this paper is to provide basic information to the public in very simple terms on how to safely and inexpensively manage wounds. This paper is directed to all nonmedical people, medical students, and other doctors who may not be content experts in this field.

## INTRODUCTION

There are many nonsurgical strategies for looking after wounds.^1^ The following is an affordable group of strategies that can easily be taught to patients and their families using simple language. This paper avoids complicated medical words so patients, medical students, and doctors all over the world can read and apply the methods we describe. Plastic surgeons have been using these simple strategies with great success for decades. This approach allows people to look after their own wounds at home at minimal financial and environmental costs with simple materials.

There are 2 basic types of wounds: (1) red, raw open wounds that are missing skin and oozing liquid and (2) closed dry wounds where skin edges are touching all along the cut and held together with stitches, staples, or glue. We will start with the care of raw open wounds.

## HOW TO CARE FOR RAW OPEN SKIN WOUNDS?

All raw wounds will heal if there is enough blood supply to the area, and if the raw tissues are not allowed to “dry and die.” Open raw wounds will heal with proper care even if there is exposed fat, bone, tendon, muscle, or joint. Red is raw; pink is healed. If the wound is red, it has lost the waterproof barrier of skin and it is an open or raw wound that oozes liquid as our bodies are 80% water. The most important part of the care of raw wounds is to keep them clean and greasy so the tissues do not dry and die. Wounds all over the body can be treated in this manner. Below, we illustrate 4 typical case examples where we use this approach in complex wounds of the foot, hand, fingertips,^2^ and face with exposed bone, cartilage, joints, and tendons. In some cases, if there are deep, open, caved in wounds, we add a vacuum assisted dressing to accelerate flattening the cave.

## DO NOT LET EXPOSED RAW TISSUES DRY AND DIE. KEEP RAW WOUNDS CLEAN AND GREASY.

### How to Clean Raw Wounds?

**Fig. 1.**
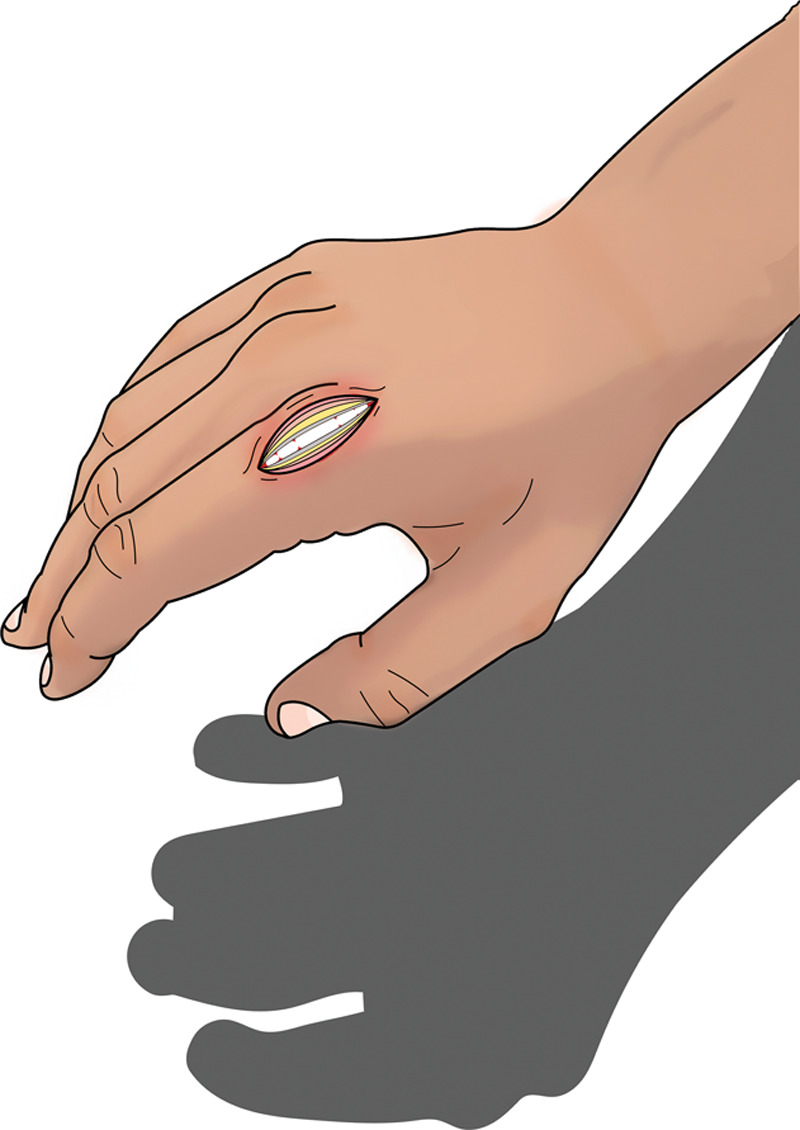
Raw wound with exposed bone, joint, and tendon.

**Fig. 2.**
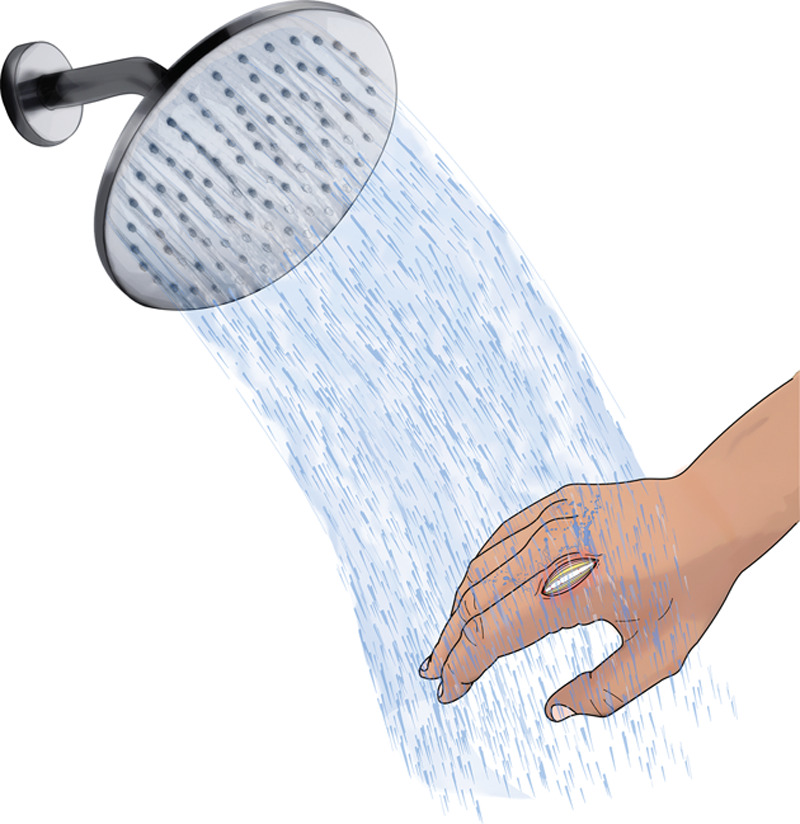
Daily shower water to rinse the wound.

**Fig. 3.**
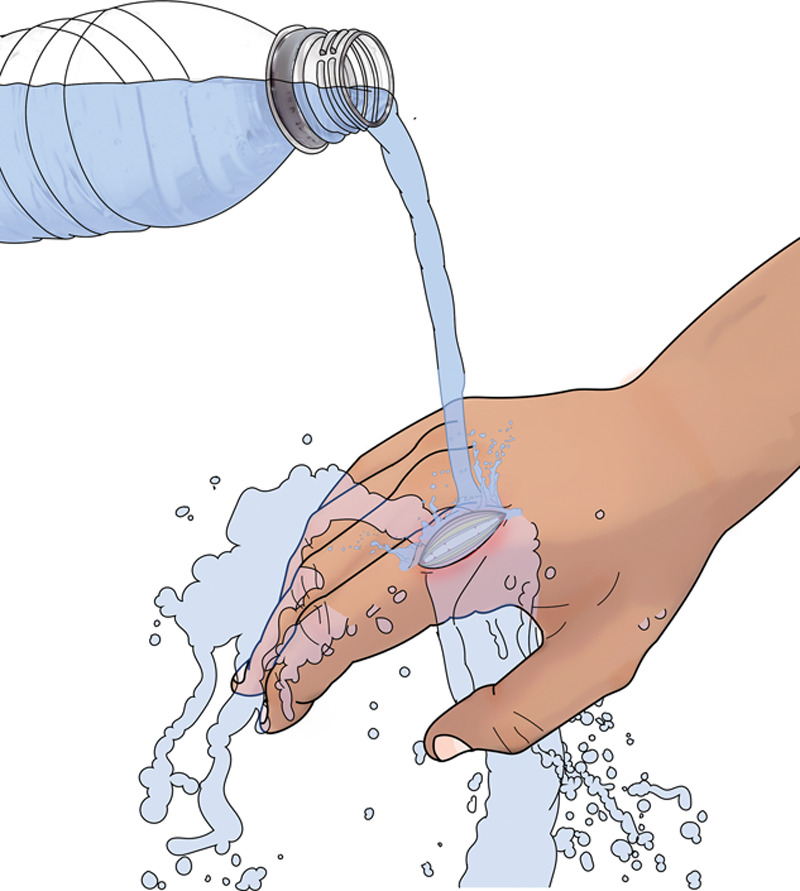
Rinse the wound with bottled water if the shower water is not clean water.

**Fig. 4.**
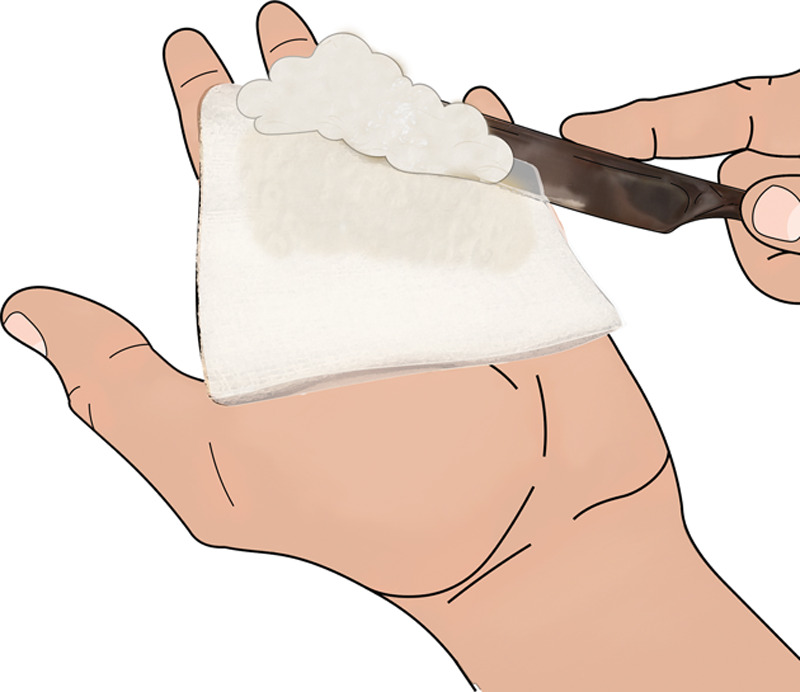
Apply Vaseline thickly to clean bandage with a clean butter knife (everything does not need to be sterile, just clean).

**Fig. 5.**
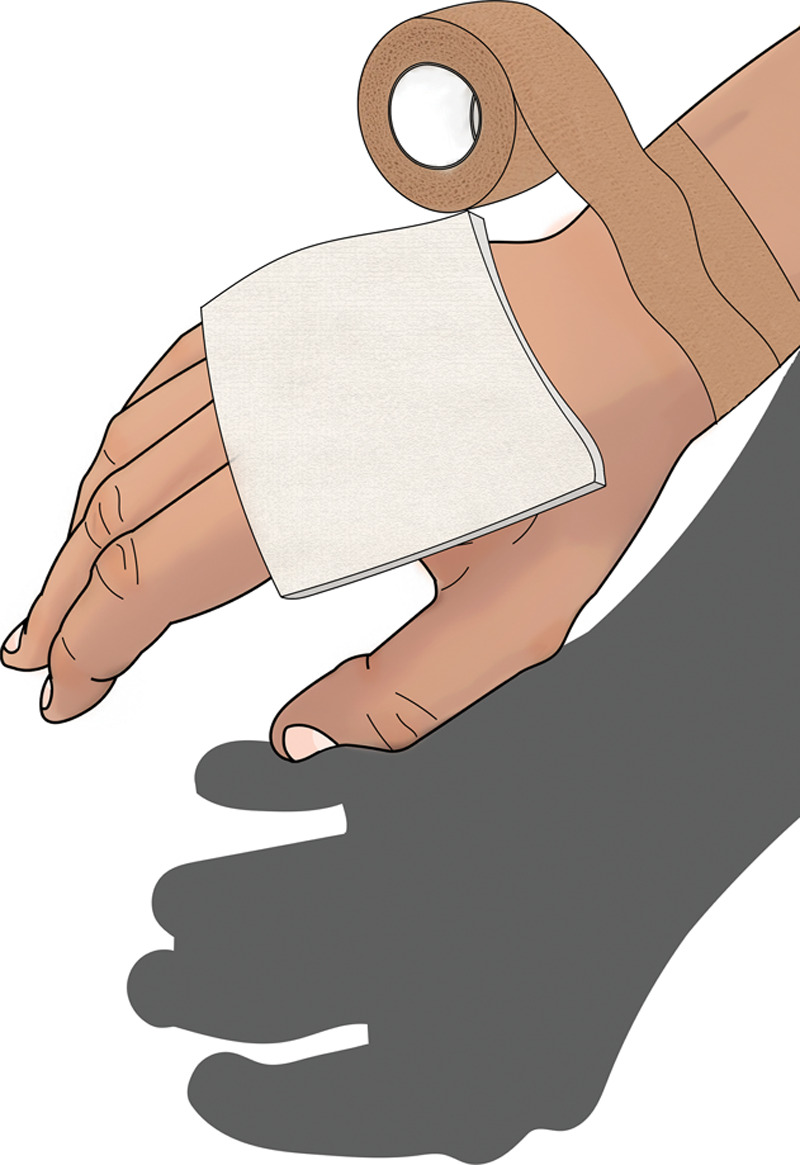
Cover wound with clean greased bandage so the wound does not dry and die.

**Fig. 6.**
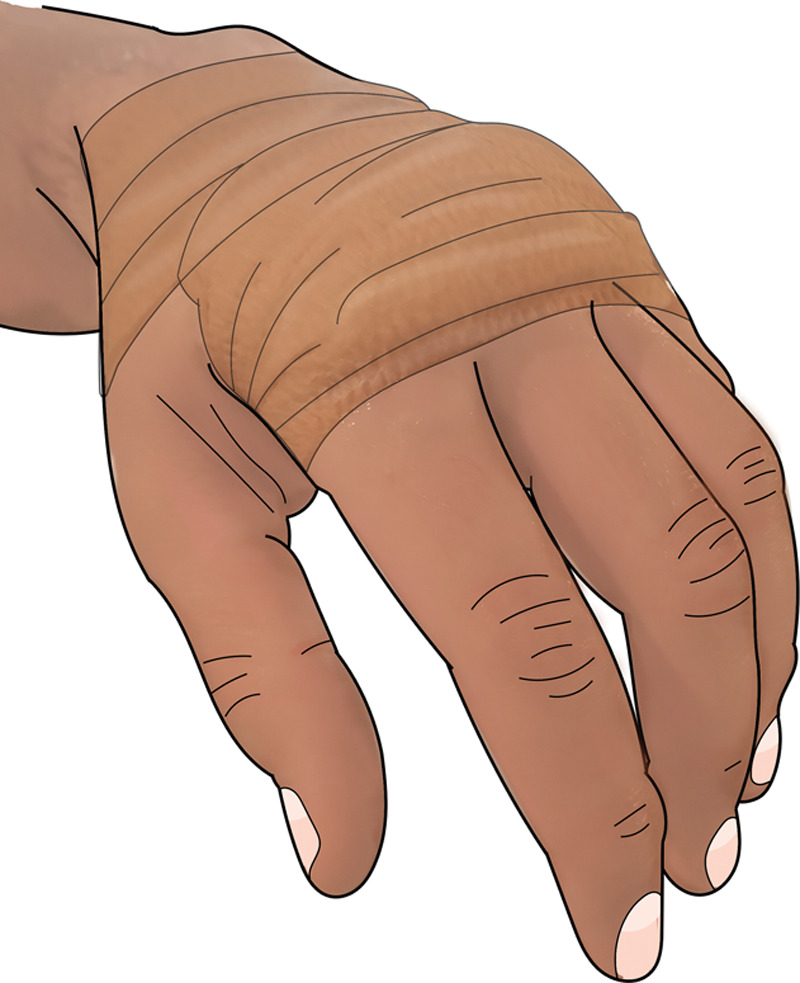
Keep the bandage secure with tape, such as Coban, so the wound stays covered with grease.

**Fig. 7.**
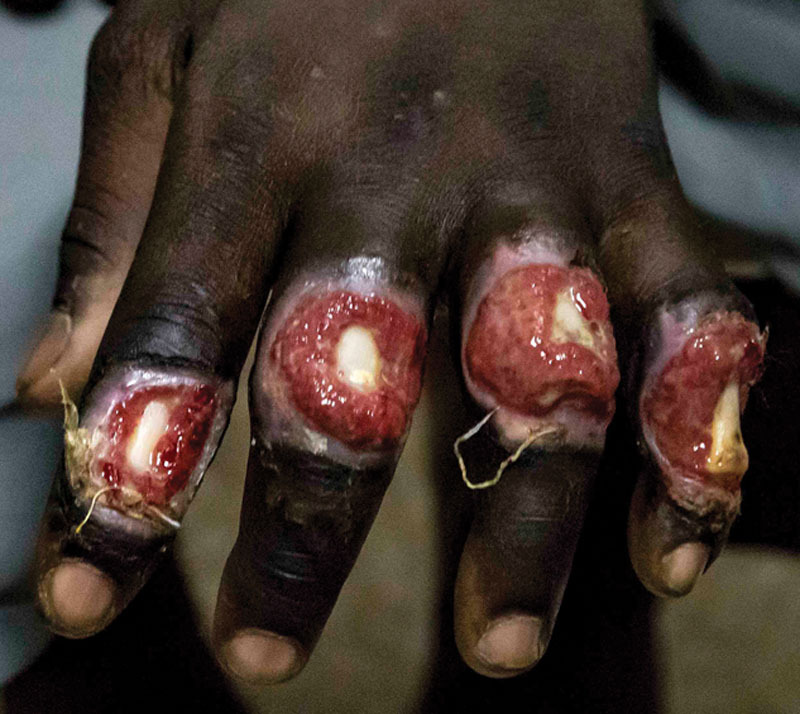
Exposed bone in 4 fingers in a patient in Ghana in 2017.

**Fig. 8.**
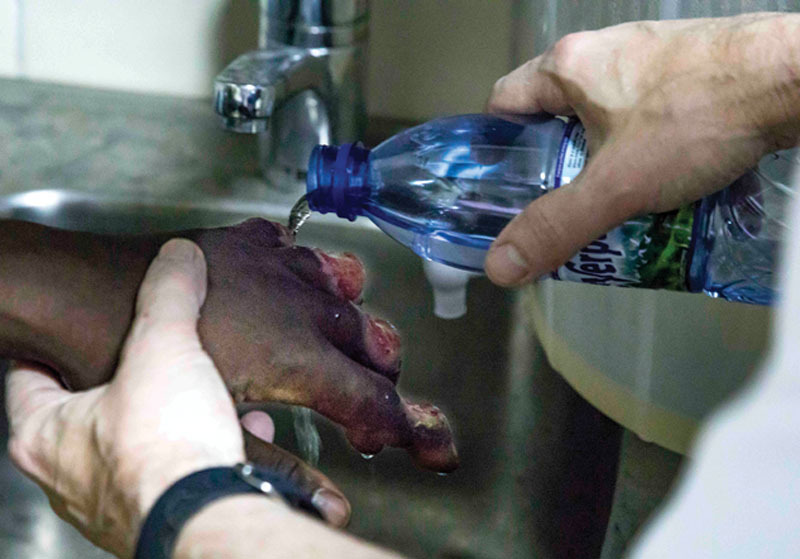
He rinsed the wound daily in the shower followed by bottled drinking water rinse.

**Fig. 9.**
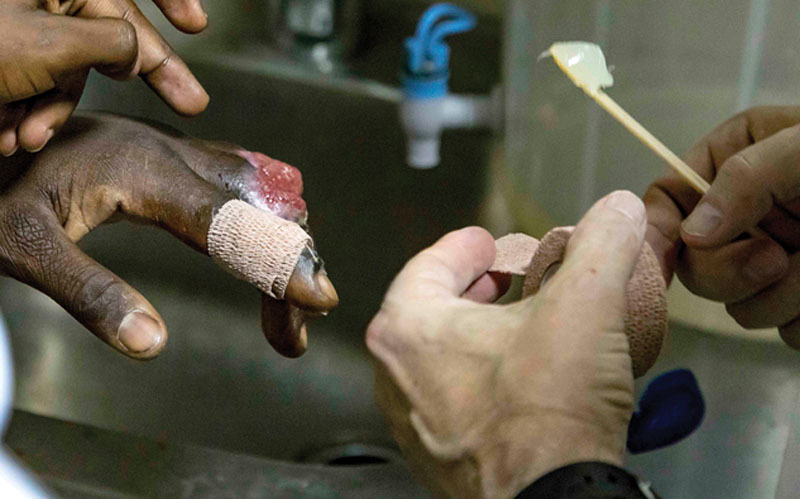
He applied Vaseline directly to Coban tape after cleaning the wound daily.

**Fig. 10.**
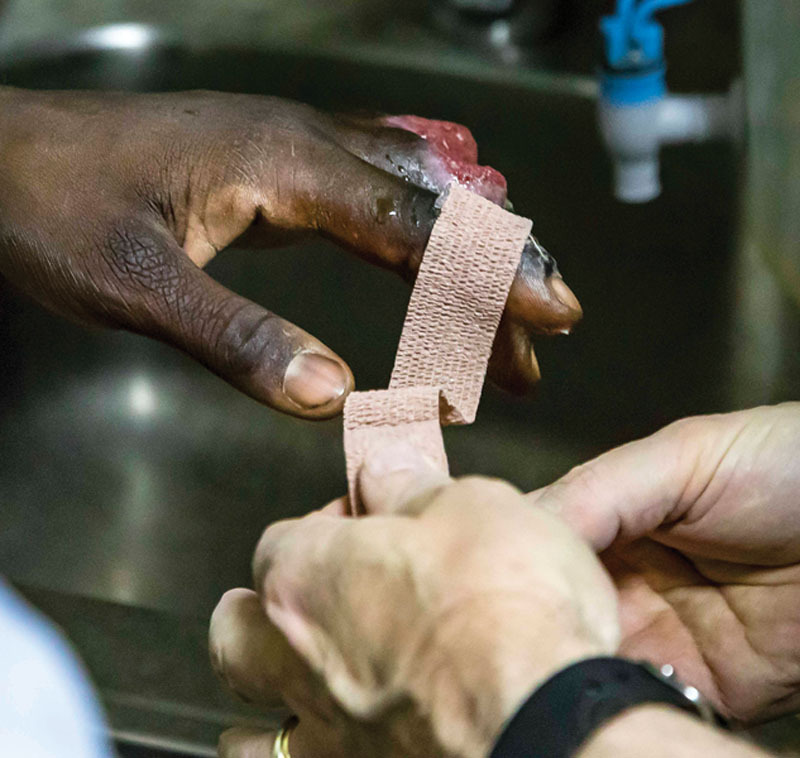
He then applied Vaseline coated Coban tape directly to the wounds to keep the wounds greasy.

**Fig. 11.**
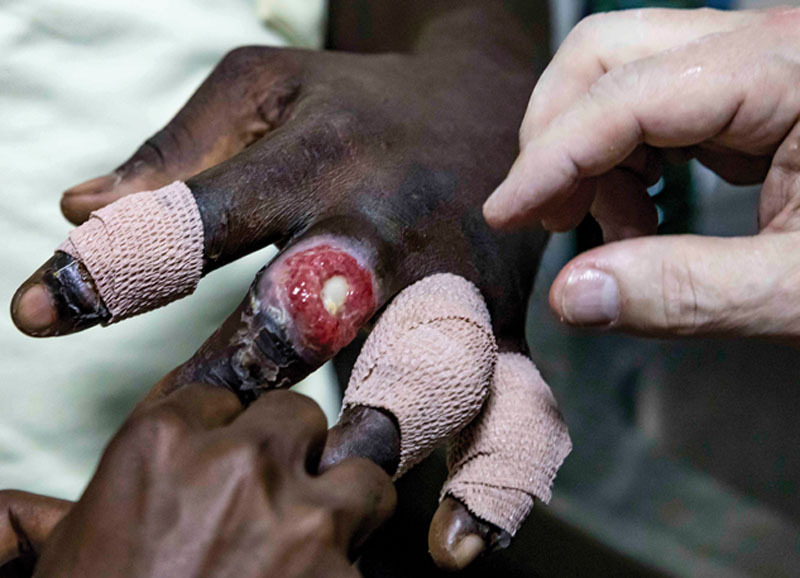
The same treatment was applied to all 4 fingers daily after the shower and bottled water rinse.

**Fig. 12.**
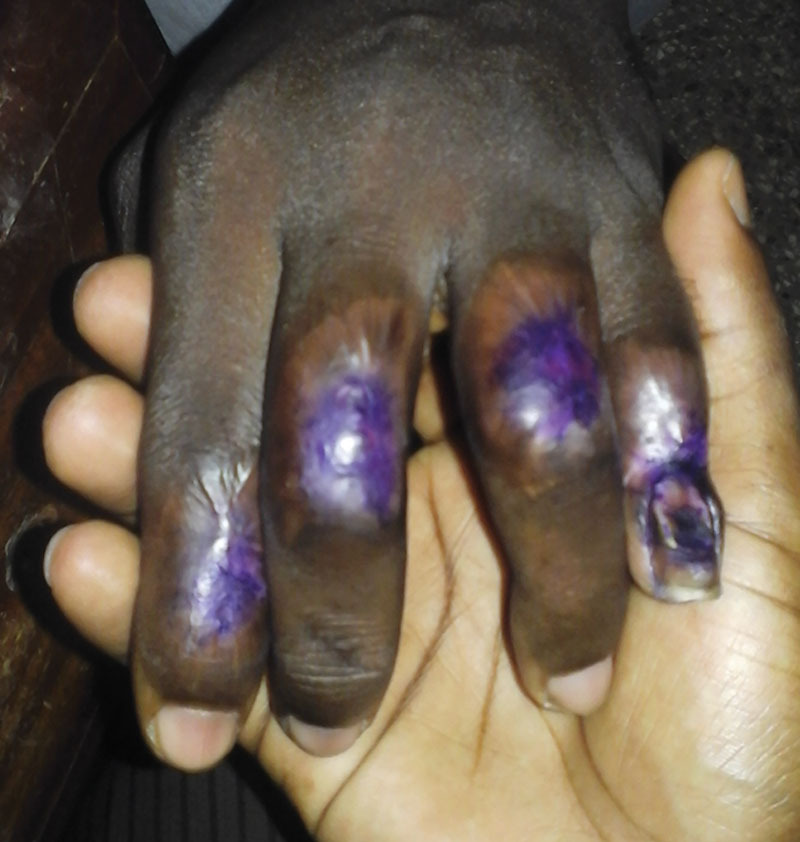
Five months later, the wounds were completely healed. Local patients in Ghana protect their scars from the sun with gentian violet, which is the purple color on the fingers. If you do not let the raw wounds dry, the tissues will not die. Skin and scar will cover the wounds with contracture and reepithelialization.

Fresh raw wounds love to be showered daily with clean water. Tap water is legislated to be almost sterile for drinking in North America. If the shower water is “dirty,” we can rinse the raw wound with bottled drinking water after letting the “dirty” shower water clean the rest of the body (Figs. 1–12) (See Video 1 [online], which displays secondary healing over exposed bone, joint, tendon, and ligaments with Coban and Vaseline in fingers; see Video 2 [online], which displays more complex facial wound managed at home with tap water, Vaseline, and clean bandages until the lip and cheek contracted in ready for forehead flap; see Video 3 [online], which displays a patient’s learning how to clean and dress wound themselves using daily shower, Vaseline, and nonsterile dressings). Sterile water or sterile saline is not necessary for cleaning wounds.^3–5^ There is no need to rub soap into a wound, but small amounts of soap or shampoo getting into a wound will not be harmful and can be rinsed out at the end of the shower. If we take a bath, we can rinse the wound with clean water at the end of the bath. Damaging nonwater liquids such as alcohol or undiluted hydrogen peroxide can kill tissue and should not be used to wash an open wound.

**Video 1.** This video displays secondary healing over exposed bone, joint, tendon, and ligaments with Coban and Vaseline in fingers.

**Video 2.** This video displays more complex facial wound managed at home with tap water, Vaseline, and clean bandages until the lip and cheek contracted in ready for forehead flap.

**Video 3.** This video displays a patient’s learning how to clean and dress wound themselves using daily shower, Vaseline, and nonsterile dressings.

### How and Why to Keep Raw Wounds Greasy?

The main rule for raw wounds is that we cannot let the tissues dry and die. Once we lose the waterproof barrier of the skin, the underlying tissues will die if they are allowed to dry. Tissues are drying, tissues are crying (they hurt, and the patient feels pain), tissues are dying.

When tissues are drying, the body tries to signal us with pain (tissues are crying in pain) that the tissues are dying. It is therefore also important that we follow “pain-guided healing.” If the wound is hurting, the dressing should be removed to make sure that there is enough grease on the wound to prevent tissues from drying.

The main active ingredient in antibiotic ointment is the grease. Vaseline or other similar inexpensive grease barriers work just as well in most cases. After washing our hands (gloves are not necessary), and after the wound has been rinsed with clean shower or bottled water, we apply a clean bandage onto which we smear a thick layer of clean Vaseline (see Video 2 [online]). The grease provides a barrier to keep the water in the raw wound, so it does not dry and die. The Vaseline does not need to be sterile, but it should be clean. It can be spread onto the bandage thickly with a clean butter knife before covering the wound with the clean bandage. The bandage does not need to be sterile.

The bigger the raw open wound, the longer it takes to heal without surgery. Most amputated finger tip wounds heal in 6–8 weeks. A larger wound, for instance, a 4-cm (1.5 inch) diameter wound of the shin missing skin may take months to heal.

### What Kind of Bandage Do We Need for Wounds?

**Fig. 13.**
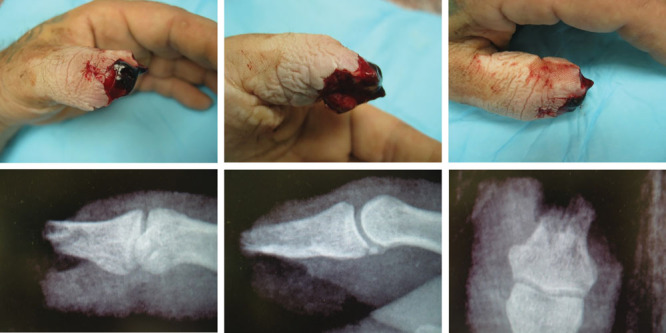
These are 3 views of an amputated thumb tip and its x-rays. One can see that the bone is clearly exposed. However, the fat, skin, blood vessels, and nerves were gradually pulled over the exposed bone over 6 weeks with wound contracture. Every day, the wound was showered and covered with Vaseline and Coban tape so the raw wound did not dry and die.

**Fig. 14.**
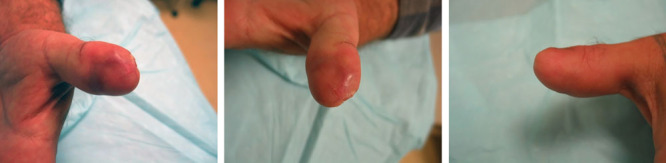
Six weeks later, the wound was healed. Moberg flap is not required in many cases. Reproduced with permission from *Plast Reconstr Surg*. 2016;137:905–906.

Dressings do not need to be sterile, just clean.^6^ Sterile dressings are expensive and unnecessary. Coban tape off the roll is a good clean dressing that can be directly applied over grease on fingers and leg wounds.^2^ Panty liners, sanitary napkins, and diapers out of the package are another good source of clean inexpensive dressings.^7^ Every day, the old dressing is removed and the patient gets in the shower to let clean water run over the wound. After the shower, grease is applied thickly to a clean bandage. That is placed on the wound to pad it, protect it, and keep it moist until tomorrow (Figs 13 and 14).

### How to Manage the Pain of a Wound?

We explain to every one of our patients that we did not spend 2 billion years evolving pain because it is bad for us. It is nature’s only way to tell us: “Hey, would you quit that? I’m trying to heal in here and you are messing it up!”

There are 2 kinds of pain with each injury or operation:

1. “The sting of the cut” which lasts for the first 1–3 days. The injured part should be elevated above the level of the heart, if possible (to help decrease swelling) and immobilized to minimize pain and set the stage for optimal healing. The patient can take simple nonnarcotic pain relievers for the sting of the wound for that time and avoid using the wounded part as much as possible. By day 2 or 3 after wounding, the sting of the cut is usually gone and the patient gets into the second kind of pain.
2. The pain of: “Gee doctor, now it only hurts when I put my wounded hand down or when I try to use it!”: it is at this point that patients should stop taking all pain killers and listen to their body. The patient should stop taking anti-pain medicine, so he knows what hurts and therefore avoids doing it. Patients should be taught not do things that cause pain in the wound because those activities can slow the healing process. This is “pain-guided healing.” It is also called common sense or instinct.

Patients should also stop taking anti-inflammatory medicine as soon as possible.^8,9^ We heal wounds with inflammation. Inflammation is a good thing. It opens closed blood vessels to increase circulation to accelerate wound healing. Patients should also be counseled to stop smoking, which greatly decreases wound blood supply and therefore slows healing.^10,11^ If the patient has diabetes, it is important to keep blood sugars under control as otherwise this can lead to a higher chance for infections and delayed healing.^12^

### Complications of Wounds that Need the Attention of a Medical Wound Specialist

The biggest problem with wounds is that they can become infected because the skin barrier to bacteria is missing. This is even more likely if there is dead tissue in the wound. Wounds that are completely pink or red inside without bad odor are usually filled with live tissue which is less likely to become infected, especially if they are kept clean and greasy so the raw tissues do not dry and die. Dead tissue can be grey or black, with a bad smell, or with draining pus, which are all signs of infection. Dead tissue is best removed by a doctor so the bacteria will have less food to eat.

Infection is frequently confused with inflammation. Inflammation around a wound is good. It is redness around the edge of the wound that is usually no wider than your thumb. It is not a sign of infection. It is the body’s way of getting more blood to the skin around the edges of the wound to bring in more healing cells that fight infection and help close the wound. Redness that extends beyond the width of a thumb (rule of thumb) may be a sign of infection. Fever and increasing pain around the wound can also be signs of infection that need the attention of a wound doctor.

### Special Wounds

#### Peripheral Vascular Disease in Smokers

Smoking makes arteries smaller and smaller over the years and eventually shuts off the blood supply to legs and feet. As a matter of fact, 1 cigarette decreases peripheral blood flow by 42%, so even 1 cigarette can have negative effects on wound healing.^12,13^ If a foot wound does not have enough blood flowing to it as occurs with peripheral vascular disease in smokers, it will not heal. The patient must understand that if he does not quit smoking, he may end up with finger or limb amputation.

#### Diabetic Foot Wounds

One of the biggest problems with diabetic foot wounds is numbness.^14^ These patients must be educated to understand that they have lost the gift of knowing when their toes or feet hurt. If they are readers, we suggest they read *The Gift of Pain* by Brand and Yancey.^15^ They must use their eyes to see wounds they cannot feel and pretend they hurt.If they could feel the wound on their foot, the pain would stop them from walking on it or dancing. They should modify their behavior to heal. For instance, they should not walk long distances to lose weight. They need to eat less instead. All of their footwear should be soft like a slipper or a thick sock.

#### Venous Insufficiency Wounds

Legs with many purple dilated veins or orange staining of the skin have incompetent veins (venous insufficiency). The most important part of managing these wounds, other than keeping them clean and greasy, is to improve the blood flow with external compressive wrapping such as stockings or elastic wrap worn at all times that the legs are down.^16^ The compression helps improve blood flow by getting blood back to the heart despite incompetent veins. The elastic compression should feel snug and comfortable. If they hurt, they are likely too tight and causing more damage. Pain-guided healing applies here and everywhere else.

#### Cancer Wounds

Some wounds exist because there is an underlying skin cancer. Suspicious wounds that just slowly get bigger for no reason need to be biopsied to rule this out.

#### Patients on Steroids

Sometimes patient are on steroid medications for other reasons. Although this may be a good solution for that particular issue, it is unfortunately not good for wound healing and can lead to delays in wound closure and increased infection rates. In addition to the principles outlined earlier that encourage daily inspection of the wound and guidelines for keeping wounds clean, it is beneficial to take over the counter vitamin A (either 25,000 international units for 3 days or 10,000 international units for 7–10 days) can help.^17–20^

## HOW TO CARE FOR CLOSED DRY WOUNDS WHERE SKIN EDGES ARE TOUCHING ALL ALONG THE CUT AND HELD TOGETHER WITH STITCHES, STAPLES, OR GLUE?

### Leave the Wounds Quiet for a Couple of Days

Do not use the body part too much and keep it elevated so gravity does not slow the blood flow, especially with arm, hand, leg, and foot wounds. Too much movement in the hours after a wound is stitched closed may generate bleeding inside the wound. This will create an internal blood clot which may slow the recovery or be a source of food for germs which may lead to infection. Get off pain medicine as soon as possible and listen to your body. “If it hurts, don’t do it.”

### Daily Shower and Bandaging

If the cut (laceration) only involves the skin, freshly sutured or stapled wounds loved to be watered gently in the shower daily as described in the “How to Clean Raw Wounds? section. (See Video 4 [online], which displays a carpal tunnel patient learning how to take pain medicine and how to shower and look after the wound after surgery). After the shower, keep the wound covered with a clean dry bandage as described in the “What Kind of Bandage Do We Need for Wounds?” section.

**Video 4.** This video displays a carpal tunnel patient learning how to take pain medicine and how to shower and look after the wound after surgery.

There are some instances where the bandage on a fresh cut should remain and not be removed by a patient. The doctor who applied the bandage will educate the patient about this. For example, if the doctor repairs a cut tendon or a broken bone under the skin, he may not want the patient to move the body part right away. He may apply a cast or splint under the bandage to stop the wound from moving. Do not remove casts or splints without the doctor’s instructions to do so.

Ointment or grease is not necessary on closed wounds because there are no red raw tissues drying and dying. There is also no value in putting vitamin E ointment on wounds to improve the appearance of scars.^21^ It does not work.

It is very important not to pick at wounds with fingernails as they can carry high concentrations of germs. It is wise to cover the wound with a clean dry bandage after the daily shower until it has quit oozing and is no longer tender to touch with freshly washed finger tips. Keeping exposed stitches and staples covered with a clean bandage will avoid them catching in material such as clothing.

## SUMMARY

Wound care does not have to be sterile, expensive, and complicated. Simple principles such as pain-guided healing, keeping raw wounds clean and greasy with a daily shower, and clean dressings are all that is required for most wounds to heal well.
