# Simple Effective Ways to Care for Skin Wounds and Incisions: Erratum

**DOI:** 10.1097/GOX.0000000000002727

**Published:** 2020-02-24

In the article, “Simple Effective Ways to Care for Skin Wounds and Incisions,” published 29 October 2019, two errors appeared in the author affiliations. First author “Don Lalonde” should be “Donald H. Lalonde,” and third author “Jeff Janis” should be “Jeffrey E. Janis.”
