# Simple Effective Ways to Care for Skin Wounds and Incisions: Erratum

**DOI:** 10.1097/GOX.0000000000003643

**Published:** 2021-05-13

In the article by Lalonde et al, “Simple Effective Ways to Care for Skin Wounds and Incisions,” published as e2471 in the October 2019 compendium *of Plastic and Reconstructive Surgery Global Open,* the authors have made a change to the Video 2 file. The initial video did not include the final result after reconstruction. The corrected final Video 2 file has been inserted to replace the incomplete version and is now available online.
